# Optimized Multi-Spectral Filter Array Based Imaging of Natural Scenes

**DOI:** 10.3390/s18041172

**Published:** 2018-04-12

**Authors:** Yuqi Li, Aditi Majumder, Hao Zhang, M. Gopi

**Affiliations:** 1 College of Information Science and Engineering, Ningbo University, Ningbo 315000, China; 2 College of Computer Science and Technology, Zhejiang University, Hangzhou 310000, China; 3 Department of Computer Science, University of California, Irvine, CA 92697-3425, USA; majumder@ics.uci.edu (A.M.); zhang.hao.alan@gmail.com (H.Z.); gopi@ics.uci.edu (M.G.)

**Keywords:** multi-spectral imaging, content independent channel selection, multispectral filter array, demosaicing

## Abstract

Multi-spectral imaging using a camera with more than three channels is an efficient method to acquire and reconstruct spectral data and is used extensively in tasks like object recognition, relighted rendering, and color constancy. Recently developed methods are used to only guide content-dependent filter selection where the set of spectral reflectances to be recovered are known a priori. We present the first content-independent spectral imaging pipeline that allows optimal selection of multiple channels. We also present algorithms for optimal placement of the channels in the color filter array yielding an efficient demosaicing order resulting in accurate spectral recovery of natural reflectance functions. These reflectance functions have the property that their power spectrum statistically exhibits a power-law behavior. Using this property, we propose power-law based error descriptors that are minimized to optimize the imaging pipeline. We extensively verify our models and optimizations using large sets of commercially available wide-band filters to demonstrate the greater accuracy and efficiency of our multi-spectral imaging pipeline over existing methods.

## 1. Introduction

Multi-channel spectral imaging using a single-camera is an efficient way to acquire spectral data. Compared with multi-camera systems that use multiple cameras with modified camera sensitivity, it is more compact and practical. Typically, the multiple different channels of the spectrum are captured (a) over multiple shots using a different color filter in front of the camera sensor for each shot; or (b) in a single shot using a mosaic of the multiple filters on the sensor forming a multiple spectral filter array (MSFA).

Traditionally, narrow band filters are used to provide a large number of channels, thereby providing higher spectral resolution assuring an accurate spectral recovery. However, this severely compromises the acquisition rate in multi-shot capture and the spatial resolution in single shot capture. Since spatial resolution is very important for most scenarios, commercially available multi-spectral cameras are multi-shot. Narrow band filters also significantly compromise light efficiency and therefore almost all multi-spectral cameras use longer exposures, thereby limiting its capture to only static scenes. Recently, use of wide-band filters has been explored to alleviate these problems by reducing the number of channels used [1]. However, the channels are usually chosen in an ad hoc manner, thereby not assuring an accurate spectral recovery. Recently, a work showed that, with channel selection, the spectral reconstruction accuracy could be improved by over 33% [2].

Previous works have shown that both man-made and natural objects or phenomena (like illumination) have smooth spectral power distribution. Statistically, their power spectrum follows a strong power law [3,4]. We exploit this statistical property to carefully select a small number of optimal wide band channels (usually 5–6) for multi-spectral imaging that can ensure an accurate spectral reconstruction when used in an MSFA for a single shot capture. The main contributions of this work are as follows.

- First, we design and develop a novel power-law prior based channel optimization method that models the various errors associated with spectral reconstruction—namely error due to estimation (reconstruction error), noise (imaging error) and demosaicing (demosaicing error). These errors depend only on the camera parameters (e.g., spectral sensitivities of channels, the MSFA pattern, the demosaicing order, and variance of the sensor noise) and not on the content. To the best of our knowledge, this is the first model for defining all the different errors in a content-independent multi-spectral imaging pipeline.
- Second, we construct an objective function that quantifies the total error using a combination of the three above-mentioned errors. Next, we use a discrete particle swarm optimization method to optimize the imaging pipeline by (1) selecting a few channels from a large set of candidate channels; (2) constructing a conducive mosaic pattern with the chosen channels on the MSFA; and (3) selecting a channel ordering during demosaicing that minimizes the objective function and hence the total error in spectral reconstruction.

## 2. Related Works

**Multispectral Imaging and Reconstruction:** a large number of the existing imaging techniques today are either bulky, or expensive and require professional calibration [5,6]. Our focus in this paper are the alternate, more compact spectral imaging techniques that are popularly used for commodity applications. There are two existing spectral imaging techniques that fit this bill—(a) compressive spectral imaging and (b) multi-spectral filter array (MSFA) based spectral imaging.

Compressive computational imaging systems randomly encode captured spectra [7], and then reconstruct multi-spectral images using compressive sensing reconstruction techniques [8,9,10]. Taking full advantages of the sparseness of spectral image data, these reconstruction techniques can recover multi-spectral images using fewer observations than conventionally needed. Furthermore, due to the high light throughput (≈0.5 [9]) of these systems, the quality of reconstructed images is robust to imaging noise. However, the sparse recovery process is time-consuming. For example, it can take minutes or even hours to reconstruct a single 512×512×31 multi-spectral frame. Therefore, it is not suitable for real-time applications, such as rendering relighted scenes, or object detection.

Contrary to compressive computational imaging, MSFA offers a very time efficient option for spectral reconstruction. Recently, it has been shown that, when using wide-band filters, MSFA based methods can be more accurate in the common case where the “photon to read noise ratios” (PRR) is not very low [11]. Therefore, various multi-channel spectral imaging system with MSFA have been designed recently. For example, Yasuma et al. [12] proposed a sub-micron pixel image sensor design with seven colors and two exposures; Sajadi et al. [13] combined a red-green-blue Bayer color filter array (CFA) with a cyan-magenta-yellow CFA in a layered CFA design to capture images with high color fidelity; Monno et al. [14] presented a high-performance multi-spectral demosaicing algorithm and utilized a single sensor with 5-channel MSFA to capture multi-spectral images. However, none of these single-shot methods focus on optimizing image pipeline parameters (like the channels selected, spatial arrangement of channels on the MSFA, demosaicing order) in a unified manner and hence cannot provide the desired spatial and spectral precision with accuracy of reconstruction.

**Figure 1:**
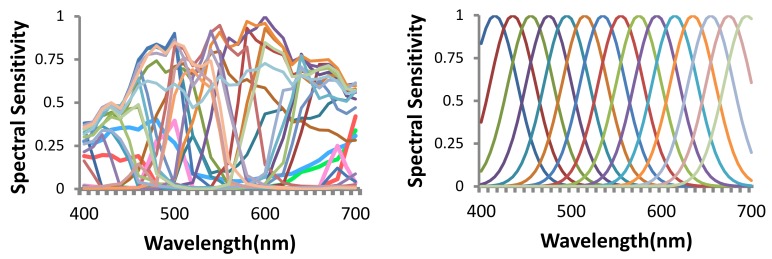
Left: commercial filter set; right: radial basis function.

**Channel Selection**: references [15,16] optimize spectral sensitivity of channels with a regular three-channel RGB Bayer color filter array (CFA) , while references [17,18] optimize channels to minimize the error in spectral recovery, but only for spectral data that have strong priors like known distribution of the captured spectral functions (e.g., daylight [19]). Such domain-specific priors yield *content-dependent* methods. More importantly, theoretically conducive spectral sensitivity of the channels are assumed (e.g., radial basis function [20,21], Fourier basis function [22]). This yields poor results in practice due to a large deviation of the sensitivities of commercially available filters from such well-behaved functions [23] (Figure 1).

In contrast, we select filters from a set of commercially available wide-band filters to create a practically feasible spectral imaging pipeline. A handful of earlier works take this route. Yasuma et al. provided a cost function to describe spectral recovery error that is optimized to select filters for the generalized assorted pixel (GAP) camera [12]. However, the design and spatial arrangement of the GAP camera lacks freedom, while impacting the quality of spectral recovery significantly. Chi et al. [1] heuristically minimize the condition number of spectral sensitivity of channels to make the spectral recovery specifically robust to noise. Other work [24] optimizing spectral reflectance targets can also be used to select channels. All these methods cannot guarantee spectral error minimization and therefore optimal spectral reconstruction. Recently, Arad et al. [2] used different three-channel combinations to reconstruct multispectral images and choose the best combination. However, it can not be extended to multi-channel selection directly since it is too time-consuming and may depend on specific spectral reconstruction method.

**Demosaicing:** demosaicing methods have been devised independent of the other elements of a spectral imaging pipeline. Interestingly, it provides a diverse view of a conducive filter array and demosaicing methods. While some prior works [25] emphasize that the spectral sensitivities of neighboring pixels should be highly correlated to minimize inter-channel demosaicing error, other works (e.g., [26]) demonstrate the benefits of completely independent demosaicing fostered by low correlation between narrow band channels. Many works [21,27] treat the channel with highest sampling rate and largest throughput as the dominant channel and complete missing values of other channels that are considered dependent on the guide channel. In addition, a recent work uses a channel-dependent demosaicing strategy for a non-channel-dominant narrow-band MSFA [28].

**Comparison with Proposed Work:** the aforementioned literature survey reveals that mutually correlated problems of channel selection, filter array design, demosaicing methods and imaging noise have always been addressed in exclusion and therefore failed to yield solutions that will be optimal in terms of accuracy and efficiency for the final output of the entire spectral imaging pipeline. Therefore, we attempt to address all these correlated issues in a unified manner and optimize them together.

## 3. Modeling Error in Spectral Recovery

Let the spectral power distribution (SPD) function of the spectrum at spatial coordinate (x,y) be s(x,y,λ). Consider a multi-spectral camera with *N* channels (N>3). Let channel *i* (1≤i≤N) have the spectral sensitivity function $m_{i}\left(\lambda \right)$ that can be obtained from specification sheets or measured using a broadband light source using some low-cost methods [29]. Let the response of channel *i* of the camera to s(x,y,λ) be $x_{i}$.

In a practical system, $x_{i}$ is comprised of ground truth, ${\tilde{x}}_{i}$, and errors, $\epsilon_{i}$. If the channel *i* at (x,y) is interpolated during demosaicing, then $\epsilon_{i}=\zeta_{i}+\delta_{i}$ where $\zeta_{i}$ denotes error due to imaging noise and $\delta_{i}$ denotes demosaicing error. For pixels where channel *i* is captured directly, $\delta_{i}=0$. Therefore,

(1) $$\begin{array}{c} \begin{array}{cc} x_{i} & ={\tilde{x}}_{i}+\epsilon_{i}=\int_{\lambda_{1}}^{\lambda_{2}}s\left(\lambda \right)m_{i}\left(\lambda \right)d\lambda +\epsilon_{i}\\ & =\int_{\lambda_{1}}^{\lambda_{2}}s\left(\lambda \right)m_{i}\left(\lambda \right)d\lambda +\zeta_{i}+\delta_{i}. \end{array} \end{array}$$

We can write the above equation using matrices considering all channels as

(2) X=Ms+ϵ=Ms+ζ+δ,

where $\left[\lambda_{1},\lambda_{2}\right]$ is the range of wavelength of visible light (e.g., 380–780 nm), *M* is a known matrix of dimension N×K where each row of *M* denotes one of the channels at a spectral resolution of *K*, *s* is the K×1 SPD function to be recovered and *X* denotes the N×1 known response vector and ϵ, ζ and δ are all N×1 error vectors.

Let us assume that the estimated spectrum $\hat{s}$ is given by multiplying *X* with reconstruction matrix *W*. Using a pseudo-inverse matrix $W^{+}=M^{T}{\left(MM^{T}\right)}^{-1}$ or a Wiener pseudo inversion $W^{+}=Corr_{s}M^{T}{\left(MCorr_{s}M^{T}\right)}^{-1}$ [30] with an approximated autocorrelation matrix $Corr_{s}$ to represent the reconstruction matrix, the recovered spectrum $\hat{s}$, is given by

(3) $$\hat{s}=WX=W\left(Ms+\epsilon \right)=WMs+W\epsilon .$$

We use the mean squared error (MSE) between the original and the recovered spectrum to describe the average error in the estimation as

(4) $$\begin{array}{c} MSE=\mathrm{Tr}\left\{E[\left(s-\hat{s}\right){\left(s-\hat{s}\right)}^{T}]\right\}. \end{array}$$

Now, replacing Equation (3) in Equation (4), we get

(5) $$\begin{array}{cc} MSE & =\mathrm{Tr}\left\{E[\left(s-WMs-W\epsilon \right){\left(s-WMs-W\epsilon \right)}^{T}]\right\}\\ & =\mathrm{Tr}\left\{E[\left(Hs-W\epsilon \right){\left(Hs-W\epsilon \right)}^{T}]\right\}, \end{array}$$

where E[.] is the expected values of a variable, H=(I−WM), and $\mathrm{Tr}\left\{.\right\}$ denotes the trace of a square matrix defined as the sum of the elements on the main diagonal. Please note that pseudo-inverse reconstruction method only provides a first approximation. Therefore, the MSE is not very accurate and only gives an upper-bound of total errors. Since the errors ϵ are independent from the spectrum *s*, $E[s\epsilon^{T}]=0$ and $E[\epsilon s^{T}]=0$. Therefore, MSE can be rewritten as

(6) $$\begin{array}{c} MSE=\mathrm{Tr}\left\{HCorr_{s}H^{T}\right\}+\mathrm{Tr}\left\{WCorr_{\epsilon }W^{T}\right\}, \end{array}$$

where $Corr_{s}$, $Corr_{\epsilon }$ are the autocorrelation matrices $E[ss^{T}]$, $E[\epsilon \epsilon^{T}],$ respectively. Interestingly, in the above equation, the first term describes the error due to spectral recovery while the latter accounts for the error due to imaging noise and demosaicing. Next, we analyze the statistical properties of the natural multi-spectral images in order to model and approximate the correlation matrix $Corr_{s}$ and $Corr_{\epsilon }$. Finally, we also describe how MSFA patterns and demosaicing interpolation affect demosaicing error and noise, respectively.

### 3.1. Spectral Characteristics of Natural Images

**Figure 2:**
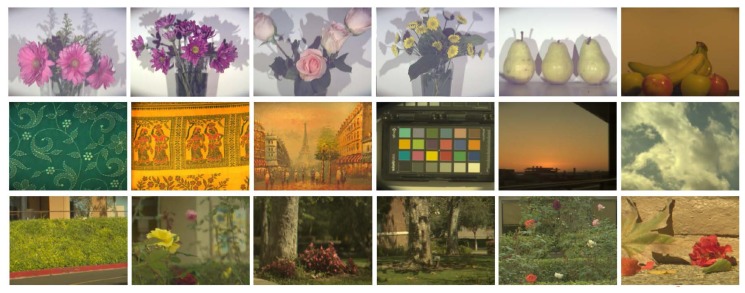
Several sample images of our natural multispectral images dataset. Each image was captured using an SOC-730 hyperspectral camera with a spatial resolution of 600 × 800 and 31 spectral measurements (400–700 nm) at each pixel.

In order to study this, we use three hyperspectral datasets—CAVE dataset, Harvard dataset, and one that we ourselves capture using a Surface Optics SOC-730 (Surface Optics Corporation, San Diego, CA, USA) camera with a 1024×1024 spatial resolution and a 2 nm (400–1000 nm) spectral resolution—this dataset contains 60 images of a sample, which is shown in Figure 2. Prior work has shown that multi-spectral images can be decomposed into Cartesian products of spectral and spatial components [31]. Therefore, we discuss them separately.

**Figure 3:**
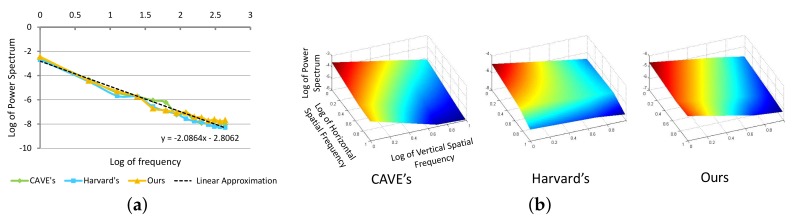
Spectral reflectance in four different data sets (Harvard’s [31], CAVE [38], and our dataset) have similar behavior after Fourier transform. (**a**) Log of power spectrum of spectral component in four different data sets; (**b**) log of power spectrum of spatial component in four different data sets.

*Spectral components*—prior works observe the following: (a) illumination and reflectance spectra of natural or man-made objects and phenomena are relatively smooth [32] and therefore frequency-limit functions can be used to approximate them [33]; (b) in addition to being smooth, these illumination and reflectance spectra also show remarkably similar energy distribution in the frequency domain (Figure 3). In fact, the surfaces formed by these distribution functions have a shape close to an exponential function with an exponent of (−2). Therefore, they follow Steven’s power law of human perception. Steven’s Law is the fundamental empirical law of human perception that most human responses to input environmental stimuli is related by a power function [34].

We illustrate the aforementioned properties in Figure 3. To eliminate the effect of varying illumination and brightness of the images, we first compute the difference spectrum Δs=s−μ, where μ is the mean value in spectral direction of multispectral images. For each Δs, we compute

(7) $$E[|\Delta s_{F}\left(k\right){|}^{2}]\propto 1/k^{2-\beta },$$

where $\Delta s_{F}\left(k\right)$ is the Fourier transform of difference spectrum Δs(λ), *k* is the frequency of spectrum, 2−β is the frequency exponent, and β clusters around 0 for natural spectra.

*Spatial components*—it is well known that the RGB real-world images, including both natural landscapes and man-made environments, also follow the Power Law [35,36] and tend to be scale invariant. This property has already been widely used in many computer graphics applications [37], e.g., exposing forgeries, imaging denoising. Interestingly, we also observe this property across different bands of real-world multispectral images (Figure 3). As in spectral components, we can show for spatial components as well that the difference band images, ΔI=I−μ, where μ is the mean of the spectral band across space, exhibits the same property as:

(8) $$E[|\Delta I_{F}\left(f,\theta \right){|}^{2}]\propto A\left(\theta \right)/f^{2-\alpha (\theta ),}$$

where $\Delta I_{F}$ is the 2D Fourier transform of difference band-images ΔI, (f,θ) is the polar coordinates of 2D frequency of band-images, A(θ) and 2−α(θ) are the scaling function and the frequency exponent function respectively for each orientation θ. Here, α(θ) clusters around 0 for natural images. Furthermore, for natural objects, the scaling function A(θ) clusters around 1; while, for man-made objects, the value of A(θ) for oblique orientation, $\theta_{o}$, is much smaller than its value for horizontal and vertical orientations, $\theta_{h}$ and $\theta_{v},$ respectively. This is due to the fact that man-made objects usually contain horizontal and vertical edges [35].

### 3.2. Modeling Recovery Error

The statistical fact that spatial and spectral components of multi-spectral images follow a power law is not dependent on any particular data set. Therefore, this provides a prior that we use to approximate recovery error $Tr\left\{HCorr_{s}H^{T}\right\}$ in Equation (6). In order to achieve this, we approximate $Corr_{s}$ by exploiting the content independent power law and the *Wiener–Khinchin theorem*. Here, every spectral wavelength sample point $s_{i}$ is treated the same. Mathematically, we assume $s_{i}=\Delta s_{i}+\mu$ (1≤i≤K) have the same distribution with the mean value μ and the standard deviation σ; therefore, $E[s_{i}^{2}]$ = $E[s_{j}^{2}]$ = $\mu^{2}+\sigma^{2}$ (1≤i,j≤K).

We approximate $Corr_{s}\left(i,j\right)$ in matrix $Corr_{s}$ using the autocorrelation value $Corr_{s}\left(i,j\right)=\mu^{2}+\eta_{1}C_{s}\left(\Delta \lambda \right)$, where Δλ is the distance between spectral band *i* and *j*, and $\eta_{1}$ is a scale factor. According to the Wiener–Khinchin theorem, the autocorrelation of a signal could be represented by using the inverse Fourier transform of power spectral function. Thus, a general form of autocorrelation value $C_{s}\left(\Delta \lambda \right)=\int \Delta s\left(\lambda \right)\Delta s\left(\lambda \pm \Delta \lambda \right)\;d\lambda$ can be described as:

(9) $$C_{s}=F^{-1}\{E[|\Delta s_{F}{\left(k\right)|}^{2}]\}.$$

Finally, our approximation of $Corr_{s}$ can be expressed as

(10) $$Corr_{s}=\mu^{2}+\eta_{1}\left[\begin{array}{cccc} C_{s}\left(0\right) & C_{s}\left(1\right) & \cdots & C_{s}\left(n-1\right)\\ C_{s}\left(1\right) & \ddots & & C_{s}\left(n-2\right)\\ \vdots & & \ddots & \vdots \\ C_{s}\left(n-1\right) & C_{s}\left(n-2\right) & \cdots & C_{s}\left(0\right) \end{array}\right].$$

Therefore, the final recovery error is estimated by $Tr\{HCorr_{s}H^{T}\}$. Compared with the methods constructing the autocorrelation matrix derived from some datasets, we use this error model because it only depends on the average intensity μ and the scale factor $\eta_{1}$. The effectiveness of the model will be verified in Section 5.1.

### 3.3. Modeling Demosaicing Error and Imaging Noise

In addition to spectral recovery error, the demosaicing error and imaging noise considerably affect the quality of the reconstruction. However, unlike the spectral recovery error, these errors are heavily dependent on (1) the design of MSFA (multi-spectral filter array), which involves the distribution and placement of the different channels in the filter array; and (2) the demosaicing technique that decides the order of the demosaicing process based on the dependency between channels or lack thereof.

**Design of MSFA:** prior works [39,40] have shown that a good MSFA pattern should have the following properties: (i) it should be spatially uniform to ensure robustness in the face of image sensor imperfections; (ii) it should be periodic to ensure efficiency of image reconstruction; and (iii) it should be neighbor consistent (each channel has the same neighbor channels) to ensure immunity to optical/electrical cross talk between neighboring pixels. Reference [40] presents a binary tree based MSFA design method that assures all the above properties and is elegant in design and implementation. Therefore, we adapt this design and customize it to suit our needs.

**Figure 4:**
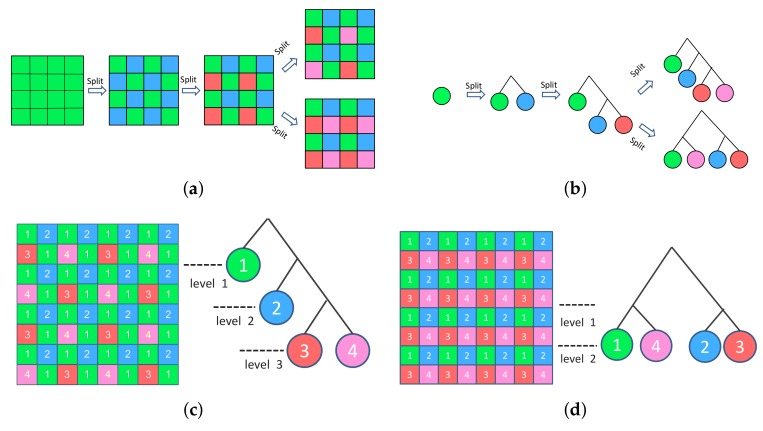
(**a**) Generation of the MSFAs. (**b**) Generation of the binary trees. (**c**,**d**) Left: two patterns of four channels MSFA; right: the binary trees of the four channels pattern have four leaves, each leaf represents a spectral channel. The four leaf nodes correspond to the four spectral channels with (**c**) sampling rate {$\frac{1}{2},\frac{1}{4},\frac{1}{8},\frac{1}{8}$} and (**d**) sampling rate {$\frac{1}{4},\frac{1}{4},\frac{1}{4},\frac{1}{4}$}.

In the binary tree based MSFA design [40], the generation of the MSFA is an iterative process of binary splitting as illustrated in Figure 4a. A parent channel evenly divides into two children channels in each splitting, increasing the number of channels by one while scaling down the sampling rate of the children created by half. Reference [40] shows that, since the MSFA patterns satisfy the three properties above, the sibling channels are exchangeable. We can use binary trees to represent such MSFA patterns (Figure 4b) where a leaf node at level *l* has a sampling rate of $2^{-l}$. Figure 4c,d shows that the patterns generated may not be unique. However, the binary tree for a particular sampling rate combination is unique.

**Demosaicing strategy:** channel-independent demosaicing completes an undemosaiced channel at a subsampled resolution without dependence of other channels. On the contrary, channel-dependent demosaicing completes an undemosaiced channel at a subsampled resolution using the content from one of more different channels. The channel dependent methods are categorized as fully dependent or partially dependent based on if they use all the channels or a subset thereof for demosaicing, respectively.

**Figure 5:**
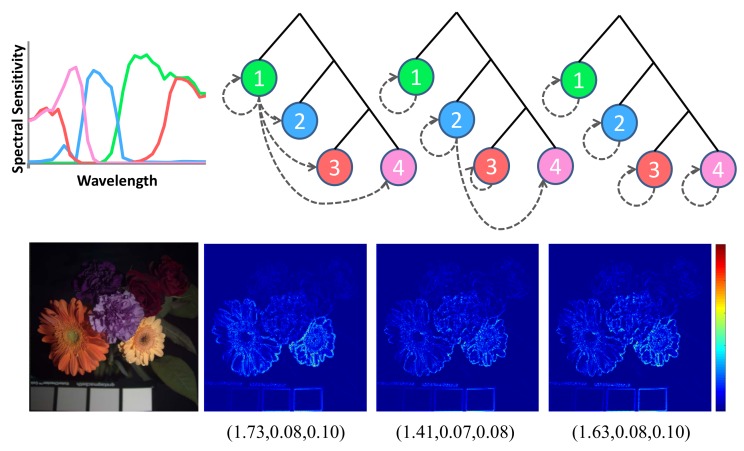
Reconstruction spectral errors of a multispectral image, and the reconstruction error statistics (mean, maxinum, standard deviation) of 18 images caused by different demosaicing strategy, the multispectral images are from CAVE dataset. Dashed arrows from channel *a* to channel *b* to denote the demosaicing of the subsampled resolution channel *b* with the guidance of the full resolution channel *a* (from left to right: “one-to-many channel-dependent demosaicing”; partial channel-dependent demosaicing; “all channel-independent demosaicing”).

Our observation reveals that neither “one-to-many channel-dependent demosaicing” [21] nor “all channel- independent demosaicing” [26] strategy are optimal (see Figure 5). Initially, we start with full resolution mosaic of the most important channel (e.g., green), we use dashed arrows from channel *a* to channel *b* to denote the demosaicing of the subsampled resolution channel *b* with the guidance of the full resolution channel *a*: self loops indicate channel-independent demosaicing of channel *b* without guidance while other arrows indicate channel-dependent demosaicing (Figure 5). It is important to note that channel selection, channel spatial arrangement, demosaicing strategy and imaging noise levels are mutually correlated. Therefore, we devise an adaptive demosaicing based on channels and imaging noise levels. In the following sections, we will model and quantify channel-independent demosaicing and channel-dependent demosaicing errors and use it for the design of the MSFA and the demosaicing method.

### 3.4. Channel-Independent Demosaicing Error

First, we consider channel-independent demosaicing for a sensor with resolution *R*. Let the set of pixels’ measuring channel *i* (at level *l* in the binary tree) directly on the sensor be $I_{i}$. Let us consider a pixel *p* and $x_{i}\left(p\right)$ denote the response of channel *i* (1≤i≤m) at the pixel *p*. If pixel $p\in I_{i}$, the response $x_{i}\left(p\right)$ is the addition of noise-free ground truth response ${\tilde{x}}_{i}\left(p\right)$ and imaging noise $\zeta_{i}\left(p\right)$. If $p\notin I_{i}$, i.e., $x_{i}\left(p\right)$ is interpolated from other directly measured pixels during demosaicing, then $x_{i}\left(p\right)$ is the sum of ${\tilde{x}}_{i}\left(p\right)$, $\zeta_{i}\left(p\right)$ and demosaicing error $\delta_{i}\left(p\right)$.

Therefore, the response $x_{i}$ at pixel *p* can be written as:

(11) $$x_{i}\left(p\right)=B_{i}\left(p\right)\left({\tilde{x}}_{i}\left(p\right)+\zeta_{i}\left(p\right)\right)+\bar{B_{i}}\left(p\right)\sum_{p^{\prime }\in P}w_{l_{i}}\left(p,p^{\prime }\right)\left({\tilde{x}}_{i}\left(p^{\prime }\right)+\zeta_{i}\left(p^{\prime }\right)\right),$$

where $B_{i}\left(p\right)$ is a binary flag that 1 for $p\in I_{i}$ and 0, otherwise, and $w_{l_{i}}\left(p,p^{\prime }\right)$ are corresponding interpolation weights, determined by both the level $l_{i}$ and the Gaussian filter used. Combining Equations (2) and (11), we find the demosaicing error and imaging noise of demosaiced pixel *p* as:

(12) $$\left\{\begin{array}{c} \zeta_{i}\left(p\right)=B_{i}\left(p\right)\zeta_{i}\left(p\right)+\bar{B_{i}}\left(p\right)\;\;\;\sum_{p^{\prime }\in P}\;\;\;\;w_{l_{i}}\left(p,p^{\prime }\right)\zeta_{i}\left(p^{\prime }\right),\\ \delta_{i}\left(p\right)=\bar{B_{i}}\left(p\right)\left({\tilde{x}}_{i}\left(p\right)-\;\;\;\sum_{p^{\prime }\in P}\;\;\;\;w_{l_{i}}\left(p,p^{\prime }\right){\tilde{x}}_{i}\left(p^{\prime }\right)\right). \end{array}\right.$$

The noise of directly observed pixels come from measurement errors and quantization errors resulting from analog to digital conversion and therefore spatially independent [41]. We use ${\bar{\zeta }}_{i}^{2}$ to denote the average directly observed noise variance in $I_{i}$. Noise variance of channel *i*, $E[\zeta_{i}^{2}]$ is a constant that can be estimated using previous method by Shimano [42] as:

(13) $$\begin{array}{cc} E[\zeta_{i}^{2}] & =\frac{1}{R}\sum_{p}\left(B_{i}\left(p\right){\bar{\zeta }}_{i}^{2}+\bar{B_{i}}\left(p\right)\;\;\sum_{p^{\prime }\in P}\;\;w_{l_{i}}^{2}\left(p,p^{\prime }\right){\bar{\zeta }}_{i}^{2}\right)\\ & =\left(2^{-l_{i}}+\left(1-2^{-l_{i}}\right)\varphi_{l_{i}}\right){\bar{\zeta }}_{i}^{2}, \end{array}$$

where $\varphi_{l_{i}}$ denotes $\sum_{p}\sum_{p^{\prime }\in P}\;w_{l_{i}}^{2}\left(p,p^{\prime }\right)$ for abbreviation. Demosaicing error of channel *i* at pixel *p* is given by the square of difference between ground truth response $x_{i}\left(p\right)$ and the interpolated value obtained from the ground truth responses of the neighboring pixels. Let us now assume that, for each pixel *p*, the square of ground truth response has the same distribution. Thus, for two pixels, *p* and *q*, p≠q, we have $E\left[{\tilde{x}}_{i}^{2}\left(p\right)\right]=E\left[{\tilde{x}}_{i}^{2}\left(q\right)\right]$. Therefore, demosaicing error variance $E[\delta_{i}^{2}]$ of channel *i* is given by the average of the variance $mean(\delta_{i}{\left(p\right)}^{2})$ of all the pixels in the image and is derived from Equation (14) as:

(14) $$\begin{array}{cc} E[\delta_{i}^{2}] & =\frac{1}{R}\sum_{p}\bar{B_{i}}\left(p\right){\left({\tilde{x}}_{i}\left(p\right)-\;\;\;\;\;\sum_{p^{\prime }\in P}\;\;\;\;w_{l}\left(p,p^{\prime }\right){\tilde{x}}_{i}\left(p^{\prime }\right)\right)}^{2}\\ & =\alpha_{l}E\left[{\tilde{x}}_{i}^{2}\right]+\sum_{p,q}^{p\neq q}\beta_{l}\left(p,q\right)E\left[{\tilde{x}}_{i}\left(p\right){\tilde{x}}_{i}\left(q\right)\right], \end{array}$$

where $\alpha_{l}$ and $\beta_{l}\left(p,q\right)$ are coefficients of $E[{\tilde{x}}_{i}^{2}\left(p\right)]$ and $E[{\tilde{x}}_{i}\left(p\right){\tilde{x}}_{i}\left(q\right)],$ respectively, in the expansion of Equation (14). We model $E[{\tilde{x}}_{i}\left(p\right){\tilde{x}}_{i}\left(q\right)]$ as $M_{i}E\left[{\tilde{I}}_{i}\left(p\right){\tilde{I}}_{i}{\left(q\right)}^{T}\right]M_{i}^{T}$, where $M_{i}$ is the known spectral sensitivity of *i* th channel. We assume a spatially independent autocorrelation matrix $E[{\tilde{I}}_{i}\left(p\right){\tilde{I}}_{i}{\left(p\right)}^{T}]$ that can be simplified to $E[{\tilde{I}}_{i}{\tilde{I}}_{i}^{T}]$. Similar to spectral variable $s_{i}$, we assume the distribution of ${\tilde{I}}_{i}$ has the mean value μ and the the standard deviation σ, where ${\tilde{I}}_{i}=\mu +\Delta {\tilde{I}}_{i}$. We use $C_{I}\left(\Delta u,\Delta v\right)=\int \;\;\int \;\Delta \tilde{I}\left(u,v\right)\;\Delta \tilde{I}\left(u\pm \Delta u,v\pm \Delta v\right)\;\;du\;dv$ to represent the ratio between correlation matrix $E[{\tilde{I}}_{i}\left(p\right){\tilde{I}}_{i}{\left(q\right)}^{T}]$ and $E[{\tilde{I}}_{i}{\tilde{I}}_{i}^{T}]$:

(15) $$E[{\tilde{I}}_{i}\left(p\right){\tilde{I}}_{i}{\left(q\right)}^{T}]=\frac{\mu^{2}+\eta_{2}C_{I}\left(\Delta u,\Delta v\right)}{\mu^{2}+\eta_{2}C_{I}\left(0,0\right)}E[{\tilde{I}}_{i}{\tilde{I}}_{i}^{T}],$$

where (Δu,Δv) is given by the absolute value of vector difference between locations of pixel *p* and *q*, and $\eta_{2}$ is a scale factor. Similar to the approximation of spectral autocorrelation matrix, $C_{I}\left(\Delta u,\Delta v\right)$ can also be approximated using Wiener–Khinchin theorem as:

(16) $$C_{I}=F^{-1}\{E[|\Delta I_{F}{\left(f,\theta \right)|}^{2}]\}.$$

Since demosaicing error is independent from imaging noise, the variance $E[\epsilon_{j}^{2}]$ in correlation matrix $Corr_{e}$ is the sum of variance of demosaicing error and imaging noise: $E\left[\epsilon_{i}^{2}\right]=E\left[\delta_{i}^{2}\right]+E\left[\zeta_{i}^{2}\right]$.

### 3.5. Channel-Dependent Demosaicing Error

In channel dependent demosaicing, we denote the guidance channel and the demosaiced channel by *g* and *r* from an upper and lower level $l_{g}$ and $l_{r}$ respectively. Prior works show that color-difference invariance in local regions is a reasonable assumption for chrominance (red/blue) channel demosaicing [43] yielding low computational complexity [44]. This color-difference invariance results in a linear demosaicing operation that is convenient for representation and computation. Therefore, we construct a demosaicing error model using the same color-difference invariance and express the response $x_{r}\left(p\right)$ of demosaiced channel *r* at position *p* as:

(17) $$\begin{array}{cc} x_{r}\left(p\right)= & {\tilde{x}}_{g}\left(p\right)+\epsilon_{g}\left(p\right)+B_{r}\left(p\right)\left({\tilde{x}}_{r}\left(p\right)+\zeta_{r}\left(p\right)-{\tilde{x}}_{g}\left(p\right)-\epsilon_{g}\left(p\right)\right)\\ & +{\bar{B}}_{r}\left(p\right)\;\;\sum_{p^{\prime }\in P}\;\;\;\;w_{l_{r}}\left(p,p^{\prime }\right)\left({\tilde{x}}_{r}\left(p^{\prime }\right)+\zeta_{r}\left(p^{\prime }\right)-{\tilde{x}}_{g}\left(p^{\prime }\right)-\epsilon_{g}\left(p^{\prime }\right)\right). \end{array}$$

From the above equation, demosaicing error variance can be transformed into a form like Equation (14) as:

(18) $$E\left[\delta_{r}^{2}\right]=\alpha_{l_{r}}E\left[{\left({\tilde{x}}_{r\backslash g}\right)}^{2}\right]+\sum_{p,q}^{p\neq q}\beta_{l_{r}}\left(p,q\right)E\left[\left({\tilde{x}}_{r\backslash g}\left(p\right)\right)\left({\tilde{x}}_{r\backslash g}\left(q\right)\right)\right],$$

where ${\tilde{x}}_{r\backslash g}$ denotes ${\tilde{x}}_{r}-{\tilde{x}}_{g}$. Here, the demosaicing error just contains the interpolation error caused in this demosaicing. The imaging noise from demosaiced channel and accumulative errors from guide channel are induced into the noise variance as:

(19) $$\begin{array}{cc} E[\zeta_{r}^{2}] & =\frac{1}{R}\sum_{p}\left[B_{r}\left(p\right){\bar{\zeta }}_{r}^{2}+\bar{B_{r}}\left(p\right)\sum_{p^{\prime }\in P}w_{l_{r}}^{2}\left(p,p^{\prime }\right){\bar{\zeta }}_{r}^{2}+\bar{B_{r}}\left(p\right)(1+\sum_{p^{\prime }\in P}w_{l_{r}}^{2}\left(p,p^{\prime }\right))\frac{E\left[\epsilon_{g}^{2}\right]-2^{-l_{g}}{\bar{\zeta }}_{g}^{2}}{1-2^{-l_{g}}}\right]\\ & =\left(2^{-l_{r}}+\left(1-2^{-l_{r}}\right)\varphi_{l_{r}}\right){\bar{\zeta }}_{r}^{2}+\frac{\left(1-2^{-l_{r}}\right)\left(1+\varphi_{l_{r}}\right)\left(E\left[\epsilon_{g}^{2}\right]-2^{-l_{g}}{\bar{\zeta }}_{g}^{2}\right)}{1-2^{-l_{g}}}, \end{array}$$

where $E[\epsilon_{g}^{2}]$ denote the error variance of guide channel *g*. Note that, in Equation (19), the noise variance of channel *r* consists of two components: (1) the imaging noise variance $\zeta_{r}^{2}$ has the same form of channel-independent demosacing (as in Equation (13)); (2) the error variance $E[\epsilon_{g}^{2}]$ of guidance channel with the coefficient decided by the level of the channel *r* and the filter used in the demosaicing algorithm. Similar to the case of channel-independent demosaicing, the error variance of demosaiced channel *r* can be calculated by $E\left[\epsilon_{r}^{2}\right]=E\left[\delta_{r}^{2}\right]+E\left[\zeta_{r}^{2}\right]$.

**Figure 6:**
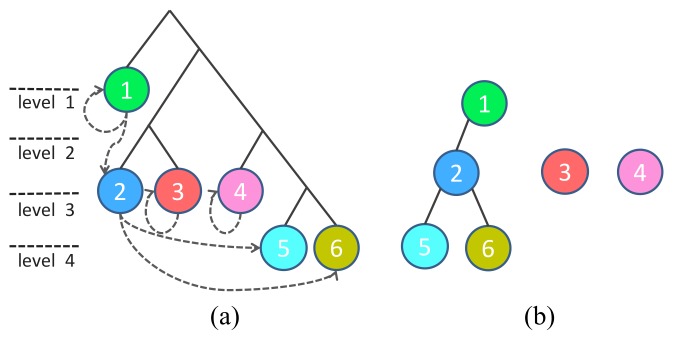
(**a**) The binary tree of a MSFA pattern and the demosaicing order; (**b**) the corresponding demosaicing forest.

The relationship between channels in demosaicing can be represented using a *Demosaicing Forest* as illustrated in Figure 6. In this forest, the channel demosaiced without guidance of other channels is the root of a tree in the forest, and a pair of parent and child nodes in a tree represents a pair of guidance and demosaiced channels in channel-dependent demosaicing. Hence, for each channel *i*, there is a unique *demosaicing chain* containing all reachable nodes from the root to channel *i*, denoted by DC(i). Intuitively, if two channels *i* and *j* come from different trees, i.e., DC(i)∩DC(j)=∅, then the two channels are independent; otherwise, the two channels would have a *lowest common chain elementk*, where the level of *k*, $l_{k}=max\left(level\left(DC\left(i\right)\cap DC\left(j\right)\right)\right)$. The errors of these two channels are then partially correlated due to sharing of the same component from the error of channel *k*. Thus, for the off-diagonal elements $E[\epsilon_{i}\epsilon_{j}]$ (the correlation between the errors of channel *i* and *j*) in correlation $Corr_{e}$, we have:

(20) $$\begin{array}{c} E\left[\epsilon_{i}\epsilon_{j}\right]=\;\;\left\{\begin{array}{c} 0,\;\;\;\;\mathrm{if}\;DC(i)\cap DC(j)=\emptyset ,\\ \frac{\left(1-2^{-l_{i}}\right){\;}^{\frac{3}{2}}\left(1-2^{-l_{j}}\right){\;}^{\frac{3}{2}}\left(E\left[\epsilon_{k}^{2}\right]-2^{-l_{k}}{\bar{n}}_{k}{\;}^{2}\right)}{\prod_{p}\left(1+\varphi_{l_{p}}\right){\;}^{-\frac{1}{2}}\prod_{q}\left(1+\varphi_{l_{q}}\right){\;}^{-\frac{1}{2}}\left(1-2^{-l_{k}}\right)},\;\;\mathrm{otherwise}, \end{array}\right.\\ s.t.\;i\neq j,p\in DC(i)-DC(k),q\in DC(j)-DC(k). \end{array}$$

Therefore, the objective function MSE is built with a few parameters: the Gaussian filter we used in demosaicing, scale factors $\frac{\eta_{1}}{\mu^{2}}$ and $\frac{\eta_{2}}{\mu^{2}}$, and imaging noise ${\bar{\zeta }}_{i}^{2}$ of each channel.

## 4. Imaging Optimization Method

In this section, to optimize reconstruction accuracy, we will seek possible combinations of channels, MSFA patterns, and demosaicing patterns that minimize the objective function (Equation (6)) with specific parameters. We propose an optimization method that selects *n* channels from *m* candidate channels (n≪m) and determines the MSFA patterns and demosaicing orders to facilitate accurate recovery of spectral reflectance of most natural and man-made objects.

Considering manufacturing complexity, we assume the number of channels, *n*, on MSFA to be relatively small (n<7). Using the binary-tree-based constraint, we know that the number of possible patterns P with *n* channels are also small. For example, for n<7, num (P)=2,3,5 when n=4,5,6. Therefore, we can enumerate every possible pattern for a specific number of channels, and calculate the optimal channel selection, the mosaic pattern, and demosaicing order for each pattern.

However, searching for an ordered subset of *n* channels from *m* candidates that would minimize the objective function is NP hard. Therefore, an exhaustive search is time-consuming. For example, with n=6 and m=100, we will need to evaluate $P\left(100,6\right)\approx 10^{12}$ permutations. Therefore, we apply a discrete particle swarm optimization (DPSO) method, detailed in Algorithm 1 to search for the optimal imaging parameters.

Particle swarm optimization [45] is a global search method that operates on a population of particles where each particle represents a candidate solution. The method moves these particles around in the search-space and updates the position and velocity of each particle iteratively.
**Algorithm 1** The Proposed DPSO Method1:**Input:** Set of candidate channels: $\left\{M_{j}\left(\lambda \right)|j=1,\ldots ,m\right\}$; Number of output channels: *n*; Pattern P.2:**Output:** Selected ordered set of *n* channels; Decided demosaicing order O.3:**Initialization:**4:Generate population $X_{i}$ and velocity $V_{i}\;\left(1\leq i\leq P\right)$5:$\left[X^{gb},O^{gb}\right]=findbest\left(X_{i}^{pb},P\right)$6:**for** *j* = 1 … G **do**7: **for** *i* = 1 … P **do**8:  $X_{i}^{pb}=f\left(M_{X_{i}},P\right)<f\left(M_{X_{i}^{pb}},P\right)?X_{i}:X_{i}^{pb}$9:  $V_{i}=w\left(V_{i-1}+c_{1}r*\left(X_{i}^{pb}⊖X_{i}\right)+c_{2}r*\left(X^{gb}⊖X_{i}\right)\right)$10:  ${\bar{V}}_{i}=reducemat\left(V_{i},p\right)$11:  $X_{i}=X_{i}⊕{\bar{V}}_{i}$12: **end for**13: $\left[X^{gb},O^{gb}\right]=findbest\left(X_{i}^{pb},P\right)\;\left(1\leq i\leq P\right)$14:**end for**15:**return** *n* ordered channels $X^{gb}$, demosaicing order $O^{gb}$;


Let the number of particles be *P* and the number of iteration be *G*. Note that *i*th particle $X_{i}$ denotes *i*th candidate solution where each solution consists of *n* ordered channel selected from *m* candidate channels, and therefore each particle $X_{i}$ is a m×1 integer vector. In vector $X_{i}$, the element with positive value *k* indicates the channel is the *k* th selected channel in pattern P, while those with 0 value indicate the channels that are not selected. The function findbest() finds the global optimal tuple of ordered channels $X^{gb}$ and the optimal demosaicing order $O^{gb}$ for the specific pattern P. The behavior of the algorithm is adjusted using four parameters: $c_{1}$, $c_{2}$, *w*, and *p*. For each iteration *j*, the algorithm maintains the global best solution $X^{gb}$ and the local best solution $X_{i}^{pb}$ of each particle. The objective function $f(M_{X_{i}},P)$ calculates the MSE of the imaging with selected channels $X_{i}$ and specific pattern P for every possible demosaicing orders and then return the minimum MSE.

Each iteration of particle *i* uses standard procedures in a basic particle swarm optimization but with modified operations. We first calculate the velocity from $X_{i}$ to $X_{i}^{pb}$ and the velocity from $X_{i}$ to $X^{gb}$ using the mutation operation ⊖. The velocity from vector *A* to *B* is defined as a differential matrix of swapping pairs from *A* to *B*. Then, the algorithm updates the velocity $V_{i}$ using weighted sum of previous velocity $V_{i-1}$, $X_{i}^{pb}⊖X_{i}$, and $X^{gb}⊖X_{i}$; here, the weighted values indicate the possibility to swapping pairs. Then, the algorithm uses the function reducemat() to retain the principal component of velocity $V_{i}$ by clipping the velocity with a threshold probability *p*; finally, it updates the solution by adding clipped velocity ${\bar{V}}_{i}$ to the current solution $X_{i}$. The adding operation $X_{i}⊕{\bar{V}}_{i}$ is defined as swapping the status(selected with index or unselected) of the *x*th channel and the *y*th channel in set $X_{i}$, where (x,y) is a swapping pair in matrix ${\bar{V}}_{i}$ [46].

## 5. Evaluation and Comparison

**Figure 7:**
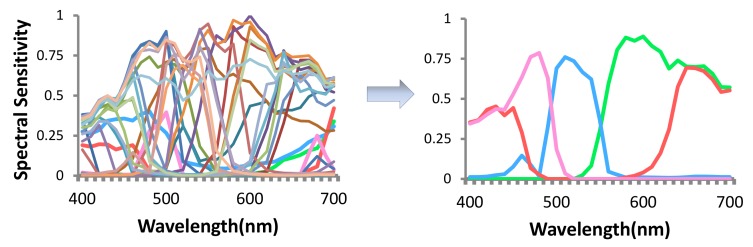
Four channels selected from a candidate set.

In this section, we evaluate our error and noise models in simulation using popular multi-spectral image datasets. Next, we compare our optimization method with GAP camera design and Chi’s filter selection method. Finally, we explore the optimal number of channels and how imaging noise level affects MSFA design and demosaicing strategy. The candidate set of channels used in simulation is constructed with spectral transmission data of 30 off-the-shelf coating color filters from http://www.rosco.com/filters (see Figure 7).

### 5.1. Error Models

We evaluate the estimated spectral recovery from our method and compare it with Chi’s [1] and Shen’s method [18]. We randomly choose 100 different channel combinations from the candidate set, each channel combination consisting of six different channels. Next, we calculate average estimated spectral recovery error only (ignoring the error due to noise and demosaicing) for each channel combination using different spectral dataset in [47], which contains natural spectral reflectances in abundance.

**Figure 8:**
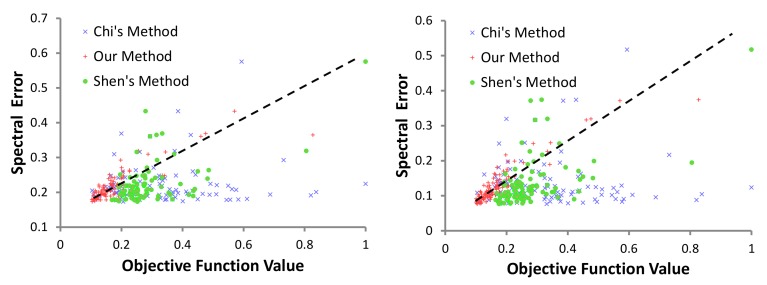
Relationship between spectral estimated error and objective function value of previous methods and our method on “natural” (**left**) and “paper” (**right**) dataset in [47]. Note the scatters of our method distribute along the black dashed line while the scatters of the previous method distribute irregularly.

Figure 8 shows the relationship between objective function values for different channel combinations and the corresponding spectral recovery error on two different datasets for Chi’s, Shen’s and our method. Note Chi’s method is content-independent, but Shen’s method uses a prior based on a large generic spectral dataset [32]. Unlike other methods, our method shows a strong linear relationship between evaluated objective function and the estimated spectral recovery error, confirming the higher accuracy of our objective function as a predictor for spectral recovery error.

**Figure 9:**
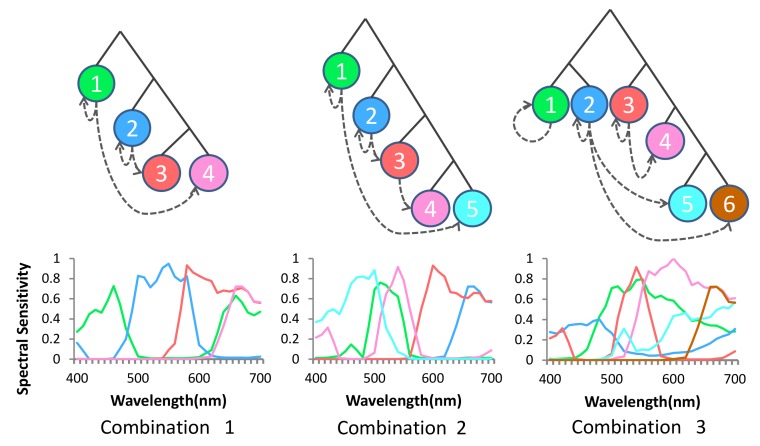
Three examples of combinations of different channels, MSFA patterns and demosaicing orders. Note that the three examples have different numbers of channels (4, 5, 6). The combinations are set casually to verify our overall model.

To evaluate our overall models, we consider three different random combination of channels, MSFA patterns, and demosaicing orders as shown in Figure 9. We adopt the bilinear interpolation method and the binary tree-based generic(BTG) demosaicing method [26] as the channel-independent demosaicing, and adopt state-of-the-art guided filter (GF) method [48] and residual interpolation (RI) method [49] as the channel-dependent demosaicing in reconstruction. In error estimation, we used scale factor $\eta_{1}=0.025$ and $\eta_{2}=0.15$, and random Gaussian noise with SNR≈50 db to the response of camera. The values of the scale factors are directly obtained from the statistics of the multispectral images in the used datasets.

**Figure 10:**
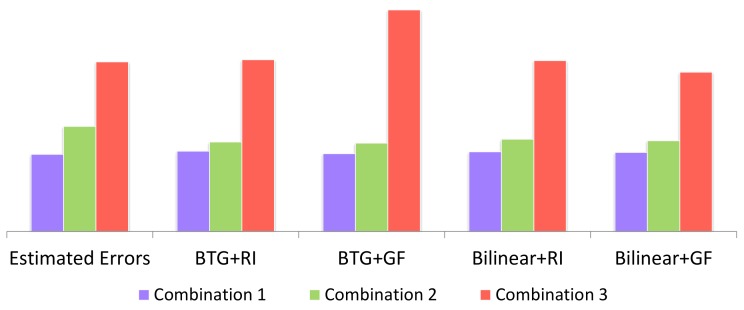
Comparison between estimated errors and actual reconstruction errors using different demosaicing methods (BTG + RI, BTG + GF, Bilinear + RI, Bilinear + GF) with the channels and patterns shown in Figure 9. The estimated errors are scaled for comparison.

The comparison between estimated errors and actual reconstruction errors using different demosaicing methods is shown in Figure 10. The actual errors are acquired by calculating the difference between ground truth and reconstructed results. It is worth noting that impact of different combination is much more than that of different demosaicing methods. Therefore, the combination is the primary factor while the methods are secondary. Our estimated errors are close to the average actual errors and illustrate the suitability of our model as a good descriptor for overall reconstruction error.

### 5.2. Comparison with Previous Methods

**Figure 11:**
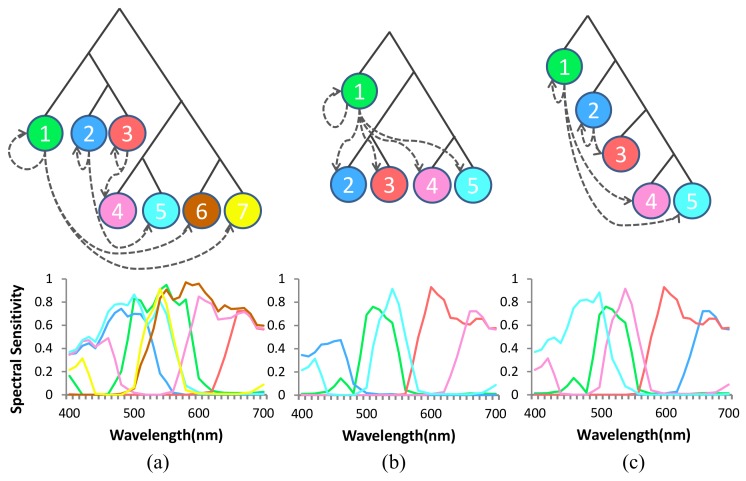
Selected channels, mosaic binary tree, and demosacking order of (**a**) GAP camera; (**b**) Chi and Monno’s method; and (**c**) our method.

To verify the effectiveness of our optimization method, we select channels from candidate channel set using our method, decide channel arrangement on MSFA pattern and demosaicing order using three methods—GAP camera [12], Chi and Monno’s method [1,14], and our method—as shown in Figure 11, and compare spectral reconstruction accuracy of our method with the other two methods on three different datasets—’CAVE’ spectral dataset [38], Harvard’s spectral dataset [31], and our dataset. Chi and Monno’s method is a combination of Chi’s channel selection method and Monno’s MSFA design and demosaicing order.

We used the demosaicing method in [26] and added random Gaussian noise to responses of camera with SNR≈50 db. To quantify the reconstruction accuracy of multispectral images, especially on edges, we use relative error |Δs(λ)| defined as:

(21) $$\left|\Delta s(\lambda )\right|=\sqrt{\frac{\int {\left(s\left(\lambda \right)-\hat{s}\left(\lambda \right)\right)}^{2}d\lambda }{\int s^{2}\left(\lambda \right)d\lambda }}.$$

Table 1 shows reconstruction error of the three optimized channel combinations in Figure 11 along with the error statistics (mean, maxinum, standard deviation). The optimized channels of our method show superior results to GAP camera’s and Chi and Monno’s results irrespective of the dataset used.

**Figure 12:**
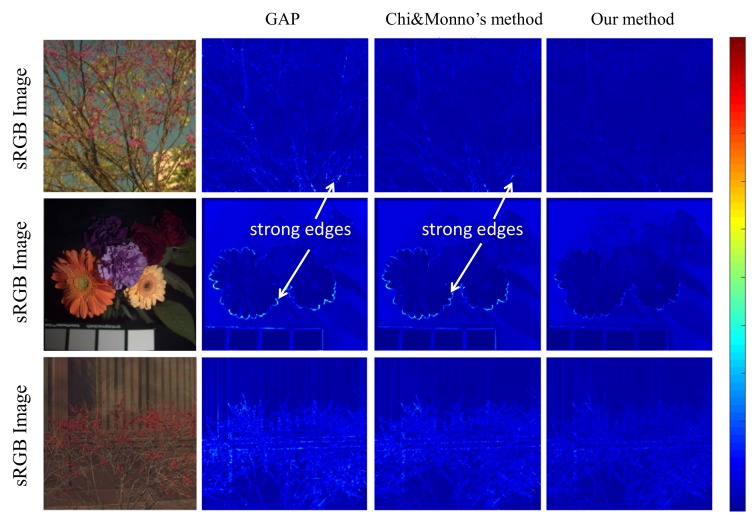
Reconstruction spectral error of multi-spectral images in our database (the 1st row), CAVE spectral database (the 2nd row), and Harvard’s database (the 3th row) using a GAP camera, Chi and Monno’s method and our mosaic camera (see Figure 11). Please zoom in and see sharp edges in gray images.

Figure 12 shows comparison of the three methods on two multi-spectral images. Since our method takes into account the demosaicing error, our estimated spectral error near sharp edges in images is significantly smaller than others.

## 6. Discussion

**Figure 13:**
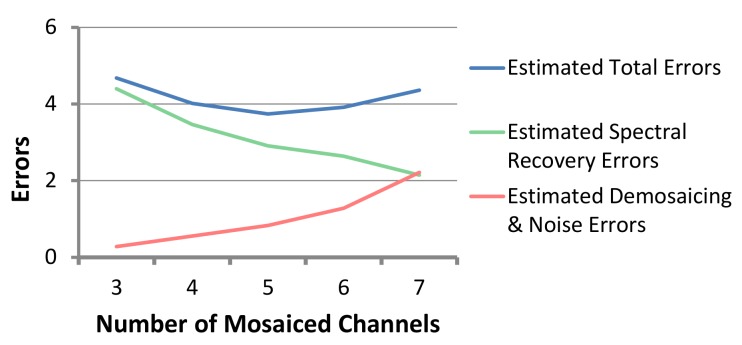
Our estimated error (spectral recovery error, demosaicing error and imaging noise) on “CAVE” spectral dataset [38] with a different number of mosaiced channels. The errors reveal the optimal number of channels to be around 5–6. Here, we use the MSFA and demosaicing method in [26].

**Optimal Number of Channels.** In order to explore the best number of mosaiced channels on MSFAs, in Figure 13, we plot the estimated error using our error model and optimization methods against the different number of channels. Note that the recovery error decreases with an increase in number of channels, while the errors caused by demosaicing and imaging noise increase with the increasing number of channels. This is expected since more channels would result in more accurate recovery while sacrificing spatial resolution for each channel. Therefore, the sweet spot is where the sum of these two curves has a minima. In Figure 13, we see that, using our candidate channels, this is around 5–6 channels.

**Figure 14:**
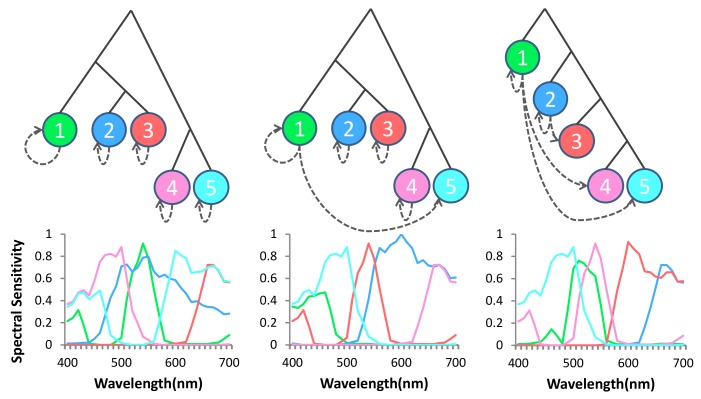
The optimized results under different noise levels (from left to right: SNR = 4, 16, 64 db).

**Behavior of Imaging Noises.** Adopting our model and optimization method, we can also explore how imaging noise affects optimal channel selection, design of MSFA, and demosaicing strategy via simulation. Figure 14 shows the optimized results under different imaging noise levels. It can be seen that, under higher noise levels, the optimized channel combination would have higher light throughput to preserve SNR of cameras (the observation is similar to previous works [13,20]). We also found that, in the presence of higher noise, the MSFA pattern tends to be more uniform, that is, the binary tree is more balanced. Furthermore, the demosaicing method tends to be more channel-independent. This is expected since, under high noise levels, the magnitude of noise is much larger than the demosaicing error, while channel-dependent demosaicing propagates noise between channels, although it can reduce demosaicing error. These conclusions provide guidelines to effectively reduce the search space in our optimization method.

## 7. Conclusions

In summary, we propose new error models for multi-spectral imaging and utilize the models to select optimal channel combination, the pattern of MSFA, and demosaicing order for multi-spectral imaging. We verified the effectiveness of our method and compared it with previous methods.

Our method can also be applied to other similar problems. For example, projection display is a dual imaging system, and, therefore, selecting few efficient primaries for a spectrally accurate display is a dual problem of our channel selection. In other areas, such as wireless sensor networking, where sensor or observation selection [50] is critical, our optimization might provide an effective solution in acceptable time.

In the future, we plan to apply our method to such varied domains, our immediate interest being in multi-spectral display. Moreover, we plan to explore the relationship between spectral sensitivity of tunable filter camera channels and accuracy of multi-spectral image registration.

## Figures and Tables

**Table 1:** Spectral reconstruction error of three optimized channels (see Figure 11) of GAP, Chi and Monno’s method, and our method.

Methods	CAVE’s Dataset			Harvard’s Dataset			Our Dataset		
	Max	Mean	Std	Max	Mean	Std	Max	Mean	Std
GAP	0.4518	0.3231	0.0880	0.2849	0.0794	0.0854	0.2602	0.1964	0.0421
Chi and Monno’s	0.4381	0.2852	0.0867	0.2498	0.0744	0.0753	0.2231	0.1880	0.0428
Ours	**0.4115**	**0.2775**	**0.0814**	**0.2196**	**0.0629**	**0.0679**	**0.1999**	**0.1586**	**0.0342**
